# An Intelligent Mechanism to Detect Multi-Factor Skin Cancer

**DOI:** 10.3390/diagnostics14131359

**Published:** 2024-06-26

**Authors:** Abdullah, Ansar Siddique, Kamran Shaukat, Tony Jan

**Affiliations:** 1 Department of Computer Sciences, Bahria University Lahore Campus, Lahore 54600, Punjab, Pakistan; 2 Centre for Artificial Intelligence Research and Optimisation, Design and Creative Technology Vertical, Torrens University Australia, Ultimo, NSW 2007, Australia; 3 School of Information and Physical Sciences, The University of Newcastle, Newcastle, NSW 2308, Australia

**Keywords:** melanoma, convolutional neural networks, deep learning, skin lesions, deep learning, machine learning, intelligent tool

## Abstract

Deep learning utilizing convolutional neural networks (CNNs) stands out among the state-of-the-art procedures in PC-supported medical findings. The method proposed in this paper consists of two key stages. In the first stage, the proposed deep sequential CNN model preprocesses images to isolate regions of interest from skin lesions and extracts features, capturing the relevant patterns and detecting multiple lesions. The second stage incorporates a web tool to increase the visualization of the model by promising patient health diagnoses. The proposed model was thoroughly trained, validated, and tested utilizing a database related to the HAM 10,000 dataset. The model accomplished an accuracy of 96.25% in classifying skin lesions, exhibiting significant areas of strength. The results achieved with the proposed model validated by evaluation methods and user feedback indicate substantial improvement over the current state-of-the-art methods for skin lesion classification (malignant/benign). In comparison to other models, sequential CNN surpasses CNN transfer learning (87.9%), VGG 19 (86%), ResNet-50 + VGG-16 (94.14%), Inception v3 (90%), Vision Transformers (RGB images) (92.14%), and the Entropy-NDOELM method (95.7%). The findings demonstrate the potential of deep learning, convolutional neural networks, and sequential CNN in disease detection and classification, eventually revolutionizing melanoma detection and, thus, upgrading patient consideration.

## 1. Introduction

Multi-type cancer is possibly the most dangerous disease and represents an immense risk to humans [1]. Among the most dangerous cancers is melanoma skin cancer, which is known to be fatal on the little chance that it is not recognized early. Early analysis can considerably lessen death rates and limit the complications of treatment. Generally, the finding system includes a biopsy, where an example is taken from the patient’s skin and inspected by a dermatologist. Accuracy of this assessment relies upon the specialist’s ability and the available apparatuses. Skin cancer is a significant public health concern worldwide. According to the latest estimates, there were approximately 8114 new cases of skin melanoma in China and 99,935 in the United States in 2022. There are also 554,274 patients in China and 554,274 in the United States [1].

The above statistics show the critical increment in daily cases, so exact determination frequently requires both experience and high-level methods to improve accuracy. Having exact instruments within reach is essential for dermatologists as they expect to accomplish precise determinations, consequently working on understanding the treatment and decreasing the number of biopsies required. This is where computerized reasoning (artificial intelligence) steps in. Specifically, profound learning, a subset of computer-based intelligence, holds an extraordinary commitment to supporting specialists in making precise conclusions and smoothing out the patient cycle [2].

As shown, deep learning innovation can speed up and improve the demonstrative cycle, in contrast to traditional manual strategies. AI offers several advantages in clinical investigation [3]. The application of deep learning makes it superior, particularly when sufficient data are available for complex diagnostic tasks [4]. One robust and deep learning procedure is convolutional neural networks (CNNs), known for their capacity to release significant elements from images [5]. CNNs have succeeded in different areas, including clinical image examination, where they assume a critical part in diagnosing conditions, like melanoma skin cancer, from images. Nonetheless, accomplishing the most significant levels of precision might require further examination and trial and error [5,6].

To address the test of diagnosing skin cancer precisely and proficiently, basic look-for arrangements provide high accuracy, speed, and patient-accommodating methodologies, possibly decreasing the requirement for intrusive techniques like biopsies. This is where deep learning becomes the most important factor. Deep learning addresses a groundbreaking frontier in the field of data science, offering unparalleled flexibility contrasted with conventional artificial intelligence [7]. Deep learning models, inspired by the human brain and its complicated neural networks, have acquired noticeable quality, especially in handling image analysis [3,8,9]. They have shown promise in illness determination and treatment in the clinical field. With the inflow of large datasets, deep learning has demonstrated its potential across various applications. In the domain of medication, it has become instrumental in tending to dangerous diseases like cancer.

Among these, melanoma skin cancer stands out and requires greater accuracy in analysis. Scientists prefer convolutional neural networks due to their widespread use in image analysis. They hold the possibility to offer a fast and accurate forecast of melanoma skin cancer, considering skin pictures, possibly decreasing the requirement for intrusive biopsies. Utilizing deep learning strategies, particularly convolutional neural networks, presents a promising path for determining melanoma and skin cancer. The adaptability and execution of deep learning, combined with the developing accessibility of large amounts of data, make it a significant resource in the basic mission of right-on-time and precise disease recognition. Scientists have proceeded to investigate and foster computational techniques that influence skin images to recognize skin cancer, which is a huge step toward better understanding consideration and possibly life-saving early conclusions.

The following section provides a literature review of multi-type skin cancer classification [10]. The rest of the paper is organized as follows. Section 2 presents a review of related work in skin lesion detection and classification. Section 3 describes the research methodology. Section 4 discusses the proposed deep Seq-CNN model. Section 5 describes the model evaluation and experimental results. Section 6 discusses various deployment aspects of the system. Section 7 presents the results and analysis. Section 8 concludes this study, and Section 9 considers future research work.

## 2. Related Work

A study detected real melanoma in dermoscopic images through deep learning, discrete coding, and SVM techniques, achieving 93.1% accuracy [11]. Another study used shared data to develop a CAD system for melanoma detection by combining handcrafted objects with deep learning. It achieved higher accuracy, specificity, and sensitivity than the ISIC 2018 dataset [11]. Research has focused on accurately assessing the risk of skin cancer, particularly melanoma, using the ABCD principle. This research proposed a method for color feature evaluation of lesion images using two datasets of dermoscopic images and one mobile-acquired dataset, which provided accuracy rates of 77.75%, 81.38%, and 93.55%, respectively. Although the methodology is successful, there are limitations, such as the reliance on machine learning and the need for broader and improved usability [12,13]. This work presented a persistent learning framework for training deep neural networks. It supports networks with up to 152 layers, achieves a 3.57% error on the ImageNet dataset, and improves object accuracy by 28% on COCO. The authors optimized a 1202-layered network, highlighting training and architectural advancement. However, overfitting is exhibited as an issue due to the complexity of neural networks [14].

The challenge of classifying skin structures in RCM images was addressed through a deep learning method combining text with neural networks and achieving an accuracy of 82%. The K-fold cross-validation confirmed the consistency of this method with an average accuracy of 81.73%. This approach is promising for the RCM tagging systems, meeting the need for video removal in situations with limited learning material [15]. The research introduced a set of DCNNs to classify dermoscopic images for skin cancer, specifically melanoma. Combining output and voting weights, the method exceeded the accuracy of individual DCNNs. The results showed an overall validation score of 0.932, indicating improved sensitivity and specificity. In summary, the DCNN class performed well in volume classification and encouraged further research to carry out improvements [16]. Deep CNNs and Transfer Learning were used to analyze clinical images of skin diseases. They use a practically identical execution as dermatologists, but the findings may not account for geographical disparities in melanoma and healthcare among various countries [17]. Precision through segmentation used the MRF hypothesis for lesion segmentation and developed a region-based segmentation [18]. Another study evaluated CAD systems for melanoma detection and examined 42 studies (24 Derm-CAD and 18 Spectro-CAD) using MSI and EIS technology. Derm-CAD had a sensitivity of 90.1%, and Spectro-CAD had a sensitivity of 92.9%, but both show a false positive rate. Specific mutations create challenges that must be optimized for clinical use. The limitations of this study include underreporting, risk of bias, and limited capacity [19].

Progressions in lesion segmentation (2018) involved remaining networks for lesion segmentation and observed improved segmentation accuracy [20]. A series of studies conducted in 2018, 2019, and 2021 compared the performance of CNNs to dermatologists in melanoma detection, achieving competitive results [21,22,23]. In (2018), the VGG16 and GoogLeNet architectures, transfer learning techniques, were used to predict skin cancer [24]. This study investigated the use of digital ex vivo confocal imaging to improve diagnostic accuracy and margin assessment during Mohs surgery for non-melanoma skin cancer (NMSC). Research has shown the success of the tumor and the surgical decisions. However, there are some limitations in terms of practicality and adoption [25]. Classifier Upgrade (2019) developed MobileNet for lesion classification. Improved accuracy, specificity, and sensitivity were observed [26]. This article presents methods for improving clinical images to diagnose eye diseases and solve noise and contrast problems. Combining filters and different tools improves image quality, as shown by parameters including PSNR, SSIM, CoC, and EPI. However, the fact that they are based on simulation and are not validated with real clinical data are major limitations [27].

Dermatology uses RCM to diagnose skin conditions that are significant for cutaneous malignancies [28]. Retinal Image Analysis (2019, 2020) has created a thorough neural technique for retinal segmentation. The research accomplished high accuracy and sensitivity [29,30,31]. AI in melanoma detection carried out AI-driven AK determination utilizing a shallow CNN in 2020. The results empowered early findings. These strategies have significantly transformed melanoma detection, evolving from an early struggle in deep learning to integrating AI into clinical practices. A literature review reveals several advances in skin cancer (especially melanoma) research using techniques such as deep learning, computer-aided design (CAD) systems, and image segmentation. However, despite the encouraging results, these studies have some limitations. These include challenges with translation models, reliance on machine learning algorithms, potential biases, and limited clinical use. Problems such as over-adaptation, geographical diversity, and practicality issues in clinical settings still need to be solved. Therefore, more research is needed to fill these gaps and overcome the limitations of previous studies. Future research should focus on developing more interpretable models, addressing biases in the dataset, performing rigorous analyses, and ensuring that solutions are feasible and possible in real clinical settings. By addressing these questions, future research may contribute to developing accurate and reliable skin cancer screening methods and, ultimately, improving the outcomes of the relationship between the patient and the clinical decision-making process.

## 3. Research Methodology

**Figure 1:**
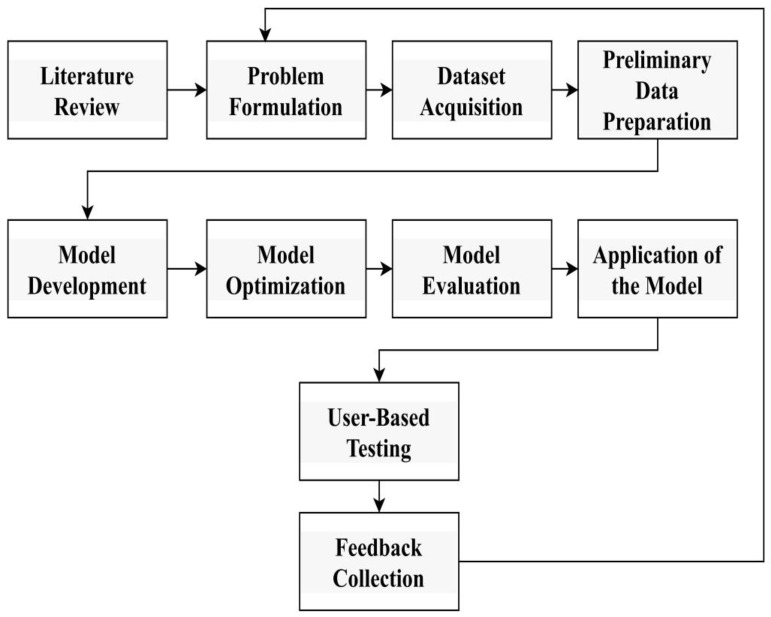
Research methodology flow.

The convolutional neural network (CNN) technique can process digital images of skin cancer to automatically extract features and patterns to indicate the presence of cancerous cells. The research begins with a comprehensive literature review, identifying inconsistencies and establishing clear objectives, as shown in Figure 1. The data were acquired by solving the class mismatch problem through data augmentation using the HAM10000 dataset. The initial data preparation includes resampling and reprocessing for sequential CNN correlation, followed by splitting into training, validation, and test cases, as shown in Figure 1. The research objective is to develop a sequential CNN model to extract features and accurately predict various types of cancer with low computational resources and cost. The optimization model uses stochastic gradient descent and various metrics to evaluate performance. The implementation of this model adds user engagement through Gradio while collecting feedback during user testing for continuous improvement and modeling unseen data recognition capability.

Graphical representation explaining the connection between research shows the impact at different levels and its contribution to the overall goal of improving the quality of skin cancer.

(a) ***Literature Review****:* A comprehensive review of the existing literature to understand research history, identify gaps, and gather insight into methods and technologies used in cancer diagnosis.
(b) ***Problem Formulation:*** Establish clear research goals and describe the research, including the type of skin cancer to be tested and the expected results.
(c) ***Dataset acquisition***

**Figure 2:**
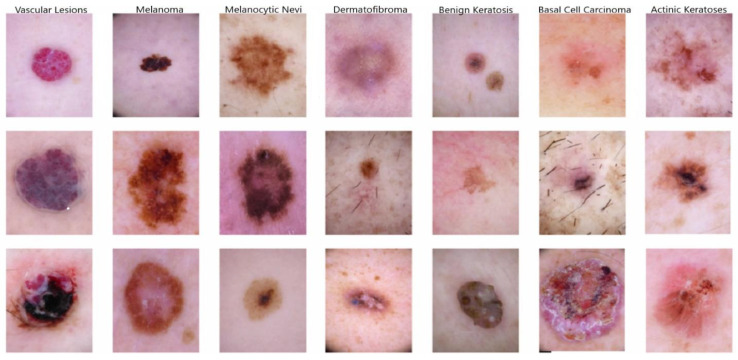
Dataset distribution of the HAM10000 skin lesion images presenting seven types of lesions, each with three samples.

The dataset used for training neural networks is the HAM10000, pivotal for automated diagnosis. HAM10000, also known as “Human Against Machine comparing with 10,000 training images”, is a standardized dataset that has served as a benchmark for the proposed approach [32]. Figure 2 presents the dataset’s actual distribution by drawing 7 columns. Each column has been labeled with a skin lesion name and has 3 samples of related lesions.

This dataset includes 10,015 dermoscopic images, primarily obtained from the ISIC archive. The HAM10000 dataset encompasses seven pigmented skin lesion classes, each addressed by a specific abbreviation as follows:

- Melanocytic nevi (NV);
- Benign keratosis-like lesions (BKLs);
- Dermatofibroma (DF);
- Vascular lesions (VASCs);
- Actinic keratoses and intraepithelial carcinoma (AKIEC);
- Basal cell carcinoma (BCC);
- Melanoma (MEL).

One notable aspect of this dataset is the substantial class imbalance, with more than two-thirds of the images belonging to the NV class. Data augmentation methods are applied to mitigate this imbalance and work on model performance, guaranteeing a more equitable distribution of images among all lesion types.

We divided our dataset into training, validation, and testing sets using different ratios to determine the optimal split. We experimented with three different splits: Split 1: 75% training, 15% validation, and 10% testing; Split 2: 70% training, 20% validation, and 10% testing; and Split 3: 60% training, 20% validation, and 20% testing. After experimenting with these splits, we chose the first split (75% training, 15% validation, 10% testing), as it best fits our model’s requirements and ensures a proper balance between the training, validation, and testing phases. We accumulated the average split rate over the three experiments and found that the chosen split is representative of the optimal ratio.

- Training data: involving 75% of the dataset;
- Validation data: making up 15% of the dataset;
- Testing data: accounting for 10% of the dataset.

To prevent data leakage, all the datasets used in the training, validation, and testing were accurately divided to support the shuffling of the split data. Splitting was performed randomly across the sample. The division was kept constant so that no testing data were used in either the training or the validation data. It was ensured that no data augmentation or preprocessing operations were performed on the test data before the final evaluation. This partitioning strategy enhances the model’s ability to be generalized. Model evaluation utilizes the ground truth labels associated with the training dataset. All the images were resized to a target size of 224 × 224 pixels for consistency and analysis.

The primary goal of this study revolves around evaluating the accuracy of skin cancer diagnosis based on dermoscopic images and leveraging the methods and strategies developed throughout this research.

- **Class-wise distribution of the HAM10000 dataset**

The class-wise circulation of images in the dataset, where each class relates to a particular skin condition.

- NV (melanocytic nevi): 6705 instances (images) have a place in this class. Melanocytic nevi are commonly benign moles or skin lesions.
- MEL (melanoma): This class comprises 1113 instances. Melanoma is a profoundly malignant type of skin cancer.
- BKLs (benign keratosis-like lesions): There are 1099 instances in this class. Benign keratosis-like lesions are skin conditions that look like keratosis but are benign (non-cancerous).
- BCC (Basal cell carcinoma): This class contains 514 instances. Basal cell carcinoma is a typical type of skin cancer that is generally non-lethal yet requires treatment.
- AKIEC (actinic keratoses and intraepithelial carcinoma): There are 327 instances in this class. Actinic keratoses and intraepithelial carcinoma address precancerous and early cancerous skin conditions.
- VASCs (vascular lesions): This class contains 142 instances. Vascular lesions involve abnormalities in veins inside the skin.
- DF (dermatofibroma): There are 115 instances in this class. Dermatofibroma is a benign skin condition portrayed by little, hard developments on the skin.

**Figure 3:**
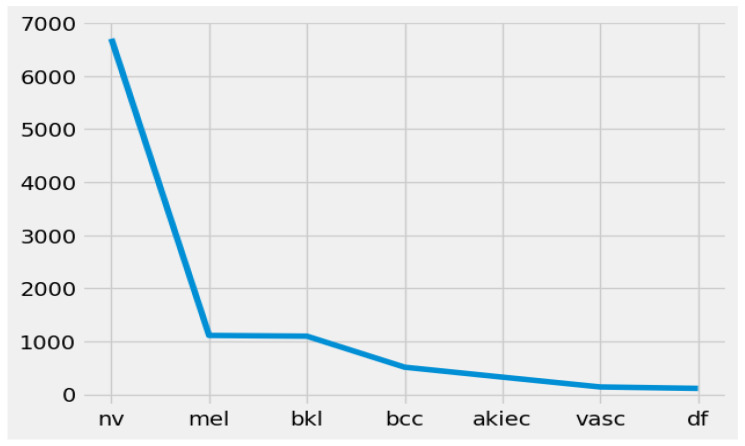
Class-wise distribution of the HAM10000 skin lesion images dataset.

This class-wise distribution, as shown in Figure 3, outlines the organization of the dataset, showing the number of images accessible for each skin condition. The plotted graph shows two axes: one axis shows the lesion class names, and the other axis shows the number of images in the relevant classes. Considering this, dissemination while working with imbalanced datasets is fundamental, as it can influence the training and evaluation of AI models. The dataset is adjusted by utilizing data resampling, which assisted with resolving potential issues brought about by class lopsidedness [32].

(d) ***Preliminary data preparation***

- Data resampling: The RandomOverSampler was used to balance the dataset by oversampling the minority class. This forestalls class imbalance issues [33].
- Data preparation for convolutional neural networks (CNNs): We reshaped the input data (XData) to have the shape (n_samples, 28, 28, 3) to match the expected input shape for a CNN that processes 3-channel (RGB) images [34].
- The pixel values were normalized by dividing by 255, which scales the pixel values between 0 and 1.
- Train, test, split: The dataset was split into training validation and testing sets using corresponding 75%, 15%, and 10% ratios. The chosen split best fits the requirements of the proposed model and ensures an appropriate balance among the training, validation, and testing phases. The shapes of the resulting training were printed into the test sets.

(e) ***Targeting Deep Neural Model for Skin Cancer Detection***

Pre-trained models, typically trained on simple images (e.g., objects and scenes), may not be well-suited for our dataset of clinical images with various skin lesions. These models may not have learned effective representations of cancerous images, which cannot be easily divided into distinct layers for processing. Therefore, the proposed sequential convolutional neural network skin cancer detection model uses several stages to translate the input data into reliable predictions. It starts by processing images from the HAM10000 dataset, scaling them to 224 × 224 pixels, and then normalizing them to a 0–1 range. Convolutional layers look for distinguishing characteristics, employing depth-wise separable convolutions and dilated convolutions to minimize computing complexity while retaining high accuracy. Following feature extraction, pooling layers (usually max pooling) lower spatial dimensions while maintaining critical data, reducing complexity [35]. The pooled feature maps were flattened into a one-dimensional vector that was processed by fully linked (dense) layers to provide an abstract representation of the features. Early halting and model checkpointing were employed to prevent overfitting, guaranteeing resilience and generalizability to new data. The final output layer uses a SoftMax activation function to transform dense layer outputs into probabilities for each class, allowing multi-class classification. Dice Loss, which is used to correct class imbalances, improves dependability in medical imaging. This sequence of data processing, feature extraction, pooling, and predicting results in a robust, efficient, and highly generalizable model for identifying and categorizing different forms of skin cancer. Using a sequential CNN instead of pre-trained models from the experiment’s outcome revealed better results on the proposed dataset. This is because complex models cause overtraining of the training data, leading to poor generalization of new unseen data. Less complex models attain feature representations that fit the dataset’s characteristics more. Using the spatial hierarchy, a sequential CNN effectively represents our dataset’s local and global features. This signifies that selecting a machine learning model that is more pertinent to the dataset characteristics is essential.

(f) ***Model optimization***

The network is trained to utilize backpropagation and optimization algorithms, like stochastic gradient descent (SGD). The loss capability, often cross-entropy, standardizes the contrast between predicted and genuine data qualities. The model is compiled utilizing sparse_categorical_crossentropy as the loss capability (reasonable for integer-encoded class labels) and the nadam optimizer. Metrics incorporate accuracy. Callbacks incorporate ModelCheckpoint to save the model with the best validation accuracy and Early Stopping to quit training if validation accuracy plateaus. The model is trained utilizing the training data and validated utilizing the testing data.

(g) ***Model Evaluation***

We used the F1 score, precision, recall, accuracy, AUC, ROC curves, sensitivity, confusion metric, and specificity [36].

(h) ***Application of the Model***

Gradio is a Python package that makes it easier to create interactive web interfaces for machine learning models. It allows us to distribute our models via web applications, allowing people to explore and interact effortlessly. Gradio eliminates the requirement for web development knowledge by making AI accessible and interactive [35].

- import Gradio as gr, and here, we imported the Gradio library, which gives tools to make basic and intuitive UIs for machine learning models.
- gr.Interface(fn = predict, inputs = image, outputs = label, capture_session = True).launch(debug = True, share = True).
- fn = predict: This specifies the function that will be used for prediction, which is the prediction function we defined earlier.
- inputs = image: This specifies that the input component of the interface is the image input we defined earlier.
- outputs = label: This specifies that the output component of the interface is the label output we defined earlier.
- capture_session = True: This captures the TensorFlow session to optimize performance.
- Launch (debug = True, share = True): This launches the Gradio interface in debug mode, allowing us to test it locally, and empowers sharing to share the interface with others.

Because the interface is created, the launch () technique is called to begin the interface, as shown in Table 1. This will make a neighborhood web server that we can access in our program. When an interface is launched, we can upload an image using the input component. After uploading, the interface will display the top predicted classes alongside their probabilities. We tried our best to design an easy-to-use interface that allows clients to upload images and obtain predictions from our machine learning model. The interface exemplifies the intricacy of preprocessing, prediction, and postprocessing, making it simple for anybody to interact with our model without composing any code.

(i) ***User-based testing via a web interface:*** *User testing is performed through a web interface or interactive interface to evaluate the usability, performance, and user experience of cancer treatment development.*
(j) ***Send feedback through questions for further improvement:*** *Address feedback from users and stakeholders through sample questions or surveys. Conduct research to identify areas for improvement, gather insight, and guide future iterations of the system.*

## 4. Proposed Model

**Figure 4:**
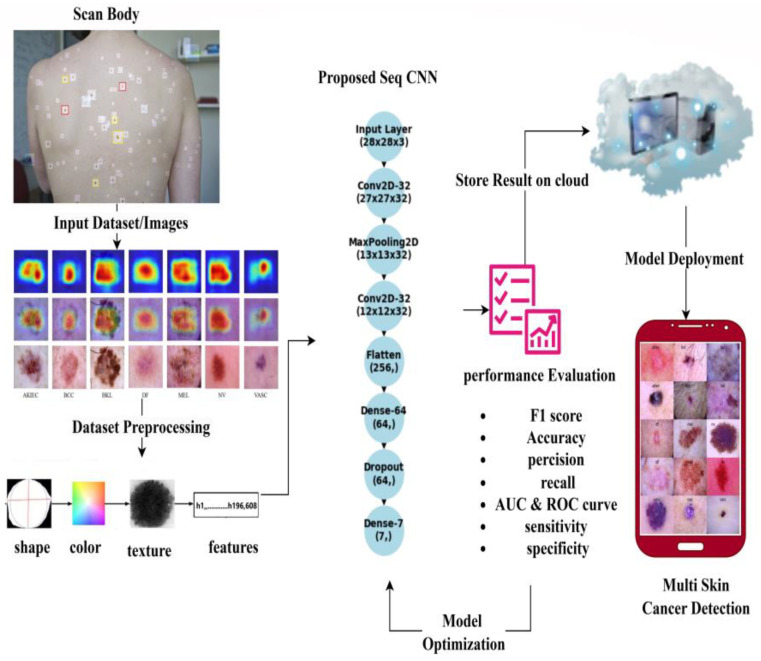
The architecture of the proposed model.

**Figure 5:**
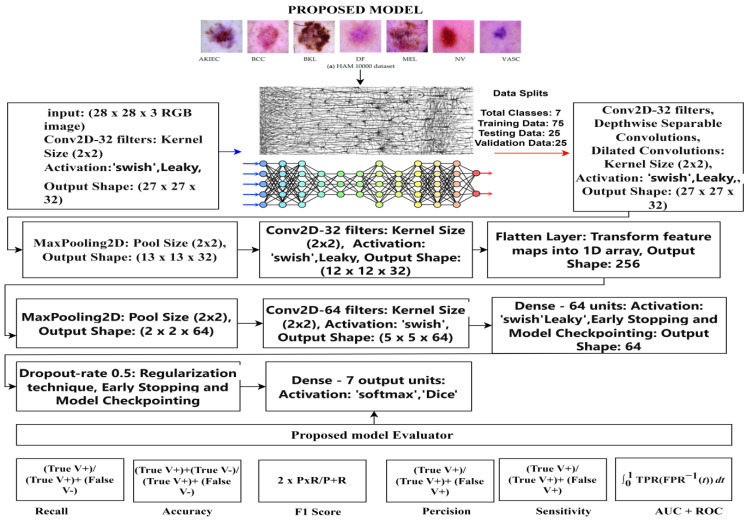
Proposed neural network layers.

A sequential convolutional neural network (CNN) is a deep learning method utilized for different undertakings, including image classification and object detection. It is classified as “sequential” because it consists of different layers stacked sequentially. Each layer in the network processes the input data and extracts relevant features before passing them to the next layer. The sequential CNN is described here, along with relevant equations to introduce the concept [17,37,38]. A CNN-based proposed model is depicted in Figure 4 and Figure 5.

(a) Input Layer

The input layer obtains the crude input data, which is ordinarily an image represented as a grid of pixel values. We should denote the input as X.

(b) Convolutional Layers

The convolutional layers are the core of CNNs, as shown in Equations (1)–(4). Depth-wise separable convolutions minimize computational costs while retaining high accuracy by separating spatial filtering and depth-based processes. Dilated convolutions extend the receptive field without increasing the number of parameters, and the model may collect more contextual data. They apply convolution operations to the input data, learning different features from the image. Swish activation is a smooth, non-linear activation function that can boost convergence and overall performance of Leaky ReLU. This form of ReLU tackles the “dying ReLU” problem by allowing small gradients for negative values, which improves robustness. The convolution operation can be represented as follows:

(1) Yi=f(Wi×X+(bi))

where

- *Yi* denotes the output feature map of the *i*-th convolutional layer. *Wi* represents the learned weights for the *i*-th convolutional filter. Additionally, *bi* is a biased term. *f* is the activation function, Leaky, and swishReLU (Rectified Linear Unit). After every convolution operation, a non-linear activation function was applied elementwise to the result.

- Pooling Layers

Pooling layers reduce the spatial dimensions of the feature maps while retaining the main data. A common pooling operation is max pooling.

(2) Yi=max−pool(Xi)

where *Yi* is the pooled feature map, and the max–poolmax–pool registers the maximum worth in a nearby region of *Xi*.

b. Fully Connected Layers

A fully connected layer is added after a few convolutional and pooling layers. Early Stopping and ModelCheckpoint are training strategies that avoid overfitting by terminating training when validation accuracy reaches a plateau and saving the best-performing model. These layers flatten the feature maps into a vector and perform traditional neural network operations.

(3) Yi=f(Wi×X+(bi))

where *Yi* is the result of the completely associated layer, *Wi* addresses the learned loads, and *bi* is the predisposition term.

c. Layer of Output

The output layer generally contains one neuron per class and employs SoftMax activation for multi-class classification. This model employs the Dice Loss function to handle class imbalance and enhance segmentation tasks, which is appropriate for medical imaging applications and provides higher performance with severely unbalanced datasets.

(4) $$Output={Softmax}_{w_{output}\times x+X_{boutput}}$$

In this equation, “*output*” addresses the anticipated probabilities for each class, where

- The *output* is the vector of anticipated probabilities, and *w_output* is the weight matrix related to the output layer. *X* is the input to the output layer. *X_boutput* is the inclination vector for the output layer, where output addresses the anticipated probabilities for each class.

## 5. Model Evaluation

The model uses the “sparse_categorical_crossentropy” loss function for multi-class classification and the “nadam” optimizer for training on augmented data to avoid class imbalance. To avoid overfitting, “ModelCheckpoint” and regularization save the best-performing model, while “EarlyStopping” halts training when validation accuracy stalls. The model trains for up to 50 epochs using 75% data for training a 15% validation set, validating against a 10% test set, and recording loss and accuracy for analysis. According to the analysis, 46 epochs were the best epochs. The architecture includes convolutional layers for feature extraction and fully connected layers for prediction, with swish activation, max pooling, and dropout used to increase generalization. This technique and the callbacks deliver strong performance in identifying different skin malignancies.

## 6. Deployment

**Figure 6:**
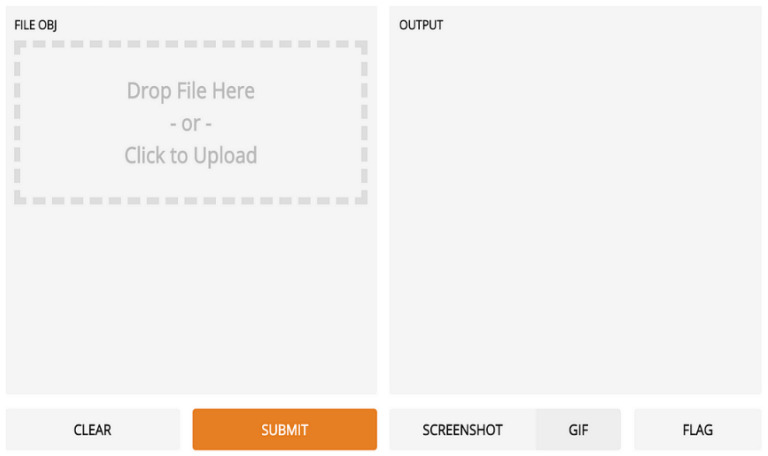
Web user interface.

We implemented our skin cancer detection algorithm using Python’s Gradio library, allowing users to input photos and obtain real-time predictions. Gradio facilitates web application creation for machine learning models, requiring only basic web programming abilities, as shown in Figure 6. To integrate with Gradio, load the library (“import gradio as gr”) and define the prediction function (“fn = predict”), as well as the input (“inputs = image”) and output (“outputs = label”) components. This approach provides a straightforward user experience, allowing for smooth interaction with the model. Setting “capture_session = True” ensures efficient session management and TensorFlow session performance, and “debug = True” improves debugging during iterative refining. Launch the web application using “gr.Interface (fn = predict, inputs = image, outputs = label)”. Launch() provides a local web server for user interaction, allowing for more efficient testing and feedback collecting. User testing is aimed at healthcare professionals and anyone interested in skin health. Users contribute images and evaluate forecasts, using feedback gathered via polls, interviews, and observation. Iterative development based on user feedback guarantees continual improvement while preserving the application’s accuracy and usability. We quickly implement the skin cancer detection model using Gradio and structured user testing, therefore contributing to medical diagnostics and machine learning.

### Discussion on the Findings of User Testing Based on Questionnaire Responses

**Figure 7:**
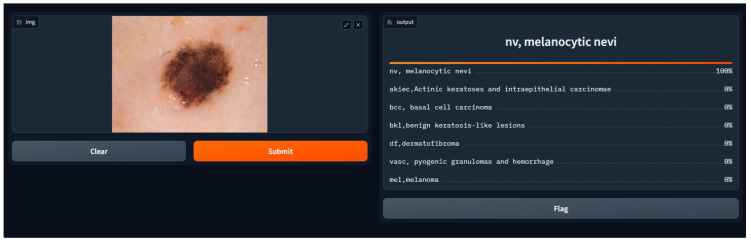
NV, Melanocytic Nevi.

**Figure 8:**
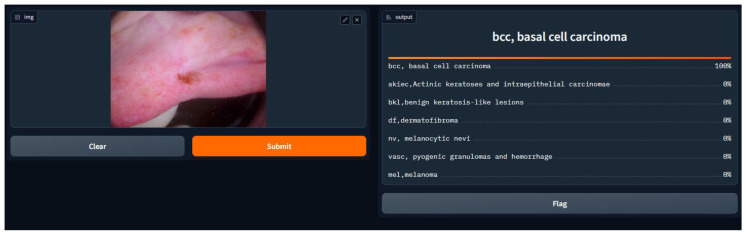
BCC, Basal cell carcinoma.

**Figure 9:**
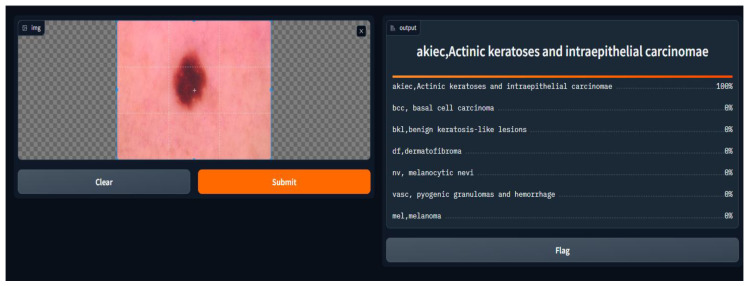
AKIEC, actinic keratoses and intraepithelial carcinoma.

**Figure 10:**
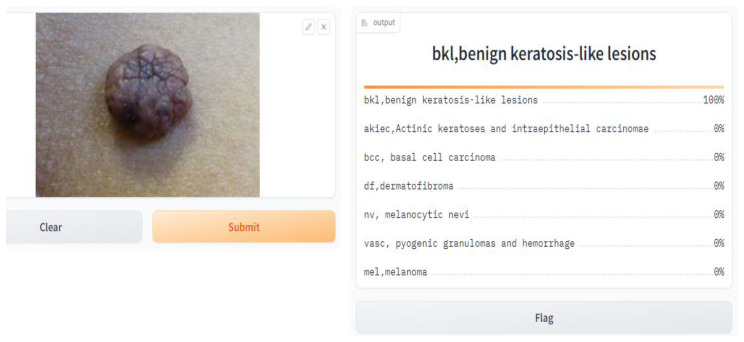
BKL, benign keratosis-like lesion.

**Figure 11:**
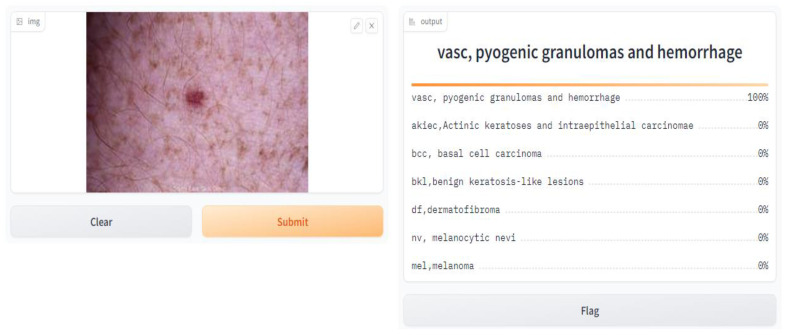
VASC, pyogenic, granulomas, and hemorrhage.

**Figure 12:**
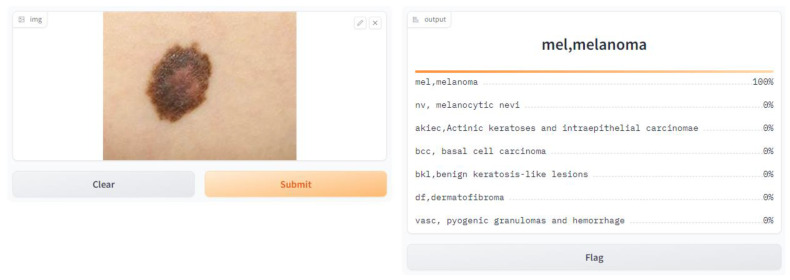
MEL, melanoma.

**Figure 13:**
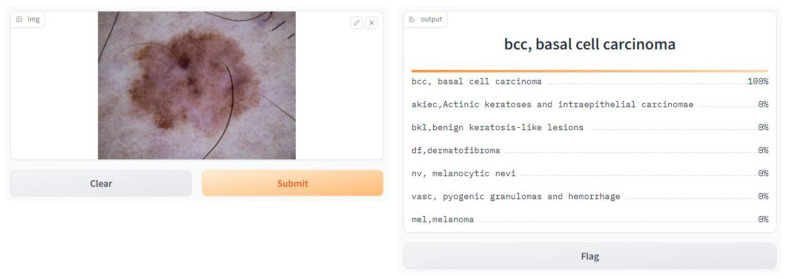
BCC, Basal cell carcinoma.

We conducted a user satisfaction survey for our skin cancer screening online application and received great input from 150 users, of which 50 were found to be patients with skin cancer. Their assessed evaluation of their skin using our tool is attached below, and discovered cases with the tool are shown in Figure 7, Figure 8, Figure 9, Figure 10, Figure 11, Figure 12 and Figure 13. The poll used a 5-point rating system to analyze several tool features and overall user experience (1—Very Dissatisfied to 5—Very Satisfied).

- Ease of use: An overwhelming 94% of users assessed the tool’s ease of use as “Very Satisfied” (5 on a 5-point scale), suggesting an intuitive and user-friendly design.
- Image upload procedure: 78% of customers were very satisfied (rating 4 on a scale) with the image upload procedure, praising its efficiency and ease.
- Accuracy and usability: The tool’s accuracy and usability garnered positive reviews, with 72% of users being “Very Satisfied” with it (5).
- Quickness: The majority of users (76%) were “Very Satisfied” (5) with the tool’s quickness in providing diagnosis findings.
- User interface: The user interface’s aesthetic attractiveness and intuitiveness satisfied 68% of users, who were “Very Satisfied” with it (5).
- Diagnostic explanation: 74% of users were “Very Satisfied” with the diagnostic explanation (5), indicating that it was clear and easy to comprehend.
- Accuracy trust: 70% of users indicated strong trust in the tool’s diagnosis (rating 4 on a scale).
- Issues or mistakes: There were no reported incidents of users having issues or mistakes during the diagnosis procedure, which is encouraging.
- Suggestions for change: Users gave useful suggestions for improving the tool’s functionality and user experience, exhibiting involvement and a desire for change.
- Privacy and security: The picture upload and data management privacy and security safeguards were well-received, with 68% of users being “Very Satisfied” with them (5).
- Overall satisfaction: 76% of users were “Very Satisfied” (5) in terms of overall satisfaction, indicating a very good reaction.
- Recommendation: Users expressed a strong willingness to suggest the product to others, with 82% being “Very Satisfied” with it (5).
- Demographics: Data on age groups, gender, and location were gathered for demographic analysis.

In summary, the user satisfaction survey reveals that users of the skin cancer screening online application had overwhelmingly good responses. High satisfaction ratings were reported in a variety of areas, emphasizing the tool’s usefulness, usability, and overall quality. User input and recommendations will be extremely helpful in improving the application’s speed and user experience.

## 7. Results and Discussion

**Figure 14:**
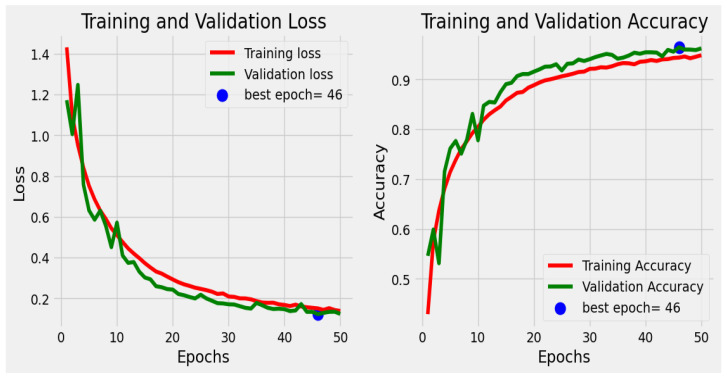
Training loss and accuracy.

**Figure 15:**
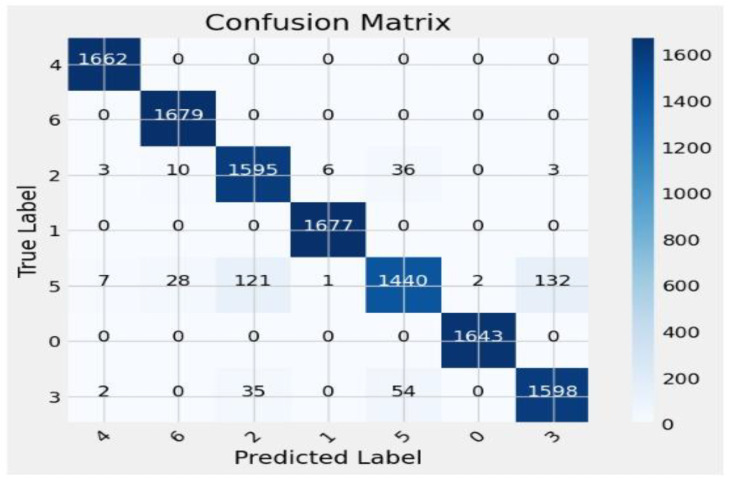
Confusion matrix of the test set.

**Figure 16:**
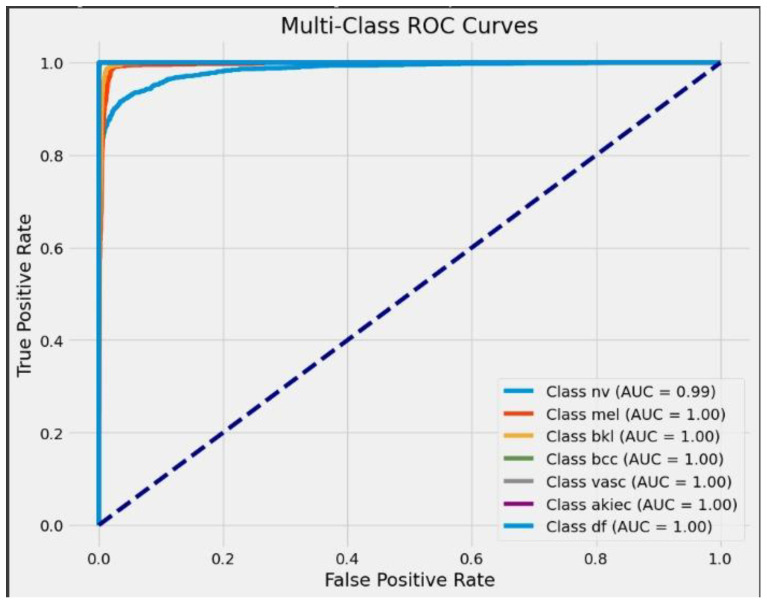
ROC curve.

The proposed sequential convolutional neural network (CNN) model was designed to classify various types of skin cancer using dermoscopic images from the HAM10000 dataset. A wide range of measures has been employed to assess its performance, such as precision, recall, F1 score, accuracy, training, and validation accuracy. These parameters are important for assessing the model’s performance and dependability in clinical settings. The precision value ranging from 0.91 to 1.00, which evaluates the model’s accuracy in predicting positive instances, is consistently high across all classes, as shown in Table 2 and Figure 14, Figure 15 and Figure 16. This demonstrates the model’s capability of reducing false positive diagnoses. Recall that the model’s capacity to recognize true positive instances performed well, indicating its competency in detecting skin cancer. The F1 score, the harmonic mean of accuracy and recall, was more than 0.88 in all classes, demonstrating a balanced approach to accuracy and recall. A confusion metric has been drawn using test set evaluation to show testing results.

**Figure 17:**
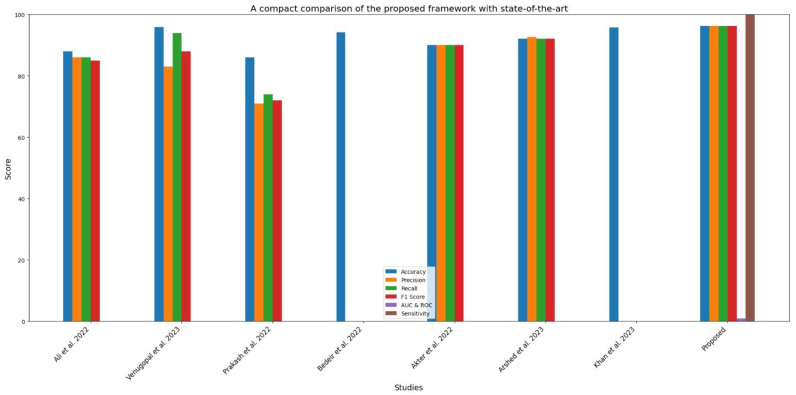
A pictorial comparison of the proposed framework with the existing methods.

The model has an overall accuracy of 96.25%, indicating a high success rate in classifying skin cancer patients. Despite the dataset’s inherent class imbalance, the constancy in both macro and weighted averages of accuracy, recall, and F1 score emphasizes the model’s reliability across classes. A high training accuracy of 98.26% indicates that the model adequately captures essential patterns in the training data, guaranteeing generalization to previously encountered scenarios [39]. We compared the proposed framework to cutting-edge models, such as CNN transfer learning (87.9%), VGG 19 (86%), ResNet-50 + VGG-16 (94.14%), Inception v3 (90%), Vision Transformers (92.14%), and the Entropy-NDOELM method (95.7%), as shown in Table 3 and Figure 17. Our model outperformed all of these approaches, illustrating the power of the sequential CNN architecture combined with stringent preprocessing procedures [40].

In Table 3 and Figure 17, the suggested model’s effectiveness can be ascribed to the use of a sequential CNN with depth-wise separable and dilated convolutions, which minimize computational cost while retaining good accuracy. The model uses early halting and model checkpointing to minimize overfitting and ensure generalization. The final output layer uses SoftMax and Dice Loss activation to handle imbalance classes, offering a stable framework for medical image applications. The model’s resilience and excellent accuracy point to its potential for improving dermatological diagnosis approaches. The framework’s capacity to reliably anticipate skin cancer diagnoses may result in improved patient outcomes and more efficient treatment techniques. Furthermore, the suggested system’s computing efficiency makes it appropriate for real-world clinical circumstances. Using randomization and pattern discovery strategies may improve diagnostic robustness. Implementing characteristics that enable the model to learn from previous experiences might result in a more intelligent diagnostic system. The suggested sequential CNN model makes considerable advances in skin cancer classification, exceeding current state-of-the-art techniques. Its excellent accuracy, precision, low computation complexity, and effective training methodologies make it an important addition to medical image diagnostics. Future work prioritized enhancing model robustness and incorporating new data sources to build a more complete approach for multi-type skin cancer diagnosis.

## 8. Conclusions

In this study, we developed a heterogeneous sequential CNN model for multi-grade skin cancer classification using majority voting and weighted majority voting, as well as a web interface for user convenience and real-time user testing of model efficacy. Deep convolution models perform exceptionally well on image datasets for skin cancer diagnosis. Several studies on categorizing skin cancer detection have already been conducted. To the best of our knowledge, no previous research has demonstrated the comparative analysis of several skin cancer types using deep sequential convolutional neural networks. Our research will assist the medical community in correctly distinguishing multi-skin cancer. A sequential CNN was used to classify seven types of skin lesions. Finally, the models’ performance is assessed using evaluation metrics such as precision, recall, F1 score, sensitivity, specificity, confusion metrics, AUC, ROC curve, and accuracy. Additionally, user testing and questionnaire responses were obtained through an online tool. These evaluations are important for assessing the model’s performance and dependability in clinical settings. Precision, which evaluates the model’s accuracy in predicting positive instances, was consistently high across all classes, ranging from 0.91 to 1.00. This demonstrates that the model can reduce false positive diagnoses. Recall that the model’s capacity to recognize true positive instances performed well, indicating its competency in detecting skin cancer. The F1 score, the harmonic mean of accuracy and recall, was more than 0.88 in all classes, demonstrating a balanced approach to accuracy and recall.

When the proposed sequential CNN was evaluated, it produced the best results, with 96.25 percent accuracy and strong positive user feedback. Therefore, our experiment reveals that the proposed sequential concurrent neural network model is more accurate than the existing models since the model does not provide biased findings. To capture changes in the shape, structure, and texture to accurately classify skin cancer images from the HAM 10,000 dataset, different sequential model features, such as Convolutional Layers, Pooling Layers, Batch Normalization, Dropout, Activation Functions (e.g., ReLU, Leaky ReLU, swish), and many other neural layers, were combined. Our research offers valuable insights for future researchers with clear observation of sequential models for further research on the skin cancer dataset. The findings show that the suggested approach outperforms dermatologists and a previously released deep learning system in classifying skin malignancies. This study found that the proposed sequential convolutional neural networks are excellent for identifying skin tumors.

## 9. Future Work

Our research on skin cancer diagnosis using convolutional neural networks (CNNs) has made significant progress, although some limitations and areas for further research have emerged. A major limitation relates to the inconsistency of the HAM10000 dataset, in which some types of skin cancer are not associated, potentially affecting the model’s predictions. Additionally, while our model performs well on test data, its ability to generalize unseen data from different populations and regions has yet to be determined. Additionally, the complexity of CNNs creates problems in explaining decision-making processes, highlighting the need to improve the interpretation of predictive models. Looking ahead, avenues for future research include data augmentation through data augmentation techniques, using hybrid or transformer-infused pre-learned CNN architectures for transfer learning, and sharing by combining other factors, such as patient populations, to better understand the skin. Additionally, describing AI technology and conducting feasibility studies with medical experts are important steps toward regulatory approval and widespread clinical use. Finally, ensuring the confidentiality and security of the solution model against attacks is important to increase the reliability and usability of AI-supported healthcare dermatology diagnostic equipment. In summary, although our study represents a significant advance in skin cancer diagnosis, addressing these limitations and pursuing future research directions are vital to realizing the full potential of AI-assisted diagnostic technology and improving patient care outcomes in dermatology practice.

## Figures and Tables

**Table 1:** Web application guide.

User Interface Web Browser	Gradio App
Gradio App	Predictions are displayed based on user input - Captures or uploads photos from the user’s camera - Local storage of images
2. Preprocessing Function	- Resizes and preprocesses the incoming picture and converts photos to a model-compatible format
3. Trained ML Model	- Neural network model trained on image data - Accepts preprocessed images as inputs - Outputs predictions for each class
4. Postprocessing Function	- Generates human-readable labels and confidence scores from model predictions
5. Display Predictions Interface	- Displays the predicted class labels and their corresponding confidence scores
6. Web Browser Output	- Renders the predictions on the user interface - Shows the class labels and confidence scores

**Table 2:** Classification report.

Classes	Precision	Recall	F1 Score	Sensitivity	Specificity	AUC	ROC	Support
0	0.99	1.00	1.00	1.00	1.00	0.99	0.99	1662
1	0.98	1.00	0.99	1.00	1.00	0.99	1.00	1679
2	0.91	0.96	0.94	1.00	1.00	0.99	1.00	1653
3	1.00	1.00	1.00	1.00	1.00	0.99	1.00	1677
4	0.94	0.83	0.88	1.00	1.00	0.99	1.00	1731
5	1.00	1.00	1.00	1.00	1.00	0.99	1.00	1643
6	0.92	0.95	0.93	1.00	1.00	0.99	1.00	1689
Accuracy			96.25%					11734
Macro avg	0.96	0.96	0.96	0.96	0.96	0.96	0.96	11734
Weighted avg	0.96	0.96	0.96	0.96	0.96	0.96	0.96	11734
Training accuracy			98. 26%					

**Table 3:** A compact comparison of the proposed framework with the state-of-the-art framework.

Article	Dataset	Preprocessing	Model	Accuracy	Classes	Image Type	Precision	Recall	F1 Score	AUC and ROC	Sensitivity
[41]	HAM10000	Yes	CNN transfer learning	87.9%	7	RGB	86%	86%	85%	--	--
[42]	HAM10000	yes	Modified EfficientNet	95.95	7	RGB	0.83	0.94	0.88	--	--
[39]	HAM10000	Yes	VGG 19	86	7	RGB	71	74	72	--	--
[43]	HAM10000	Yes	ResNet-50 + VGG-16	94.14%.	7	RGB	No	No	No	--	--
[44]	HAM10000	Yes	Inception v3	0.90	7	RGB	0.90	0.90	0.90	--	--
[45]	HAM10000	Yes	Vision transformers (RGB images)	92.14%	7	RGB	92.61%	92.14%	92.17%	--	--
[46]	HAM10000	Yes	Entropy-NDOELM algorithm	95.7%	7	RGB	No	No	No	--	--
**Proposed**	**HAM10000**	**Yes**	**Sequential convolutional neural network**	**96.25**	**7**	**RGB**	**96.27**	**96.23**	**96.23**	**0.96**	**100**

## Data Availability

Data are available in a publicly accessible repository. The data presented in this study are openly available in the Kaggle Repository at doi:10.1038/sdata.2018.161 (2018), Ref. [32].

## Abbrevations

**Acronyms**	**Expanded Form**	**Acronyms**	**Expanded Form**
ABCD	Asymmetry, Border, Color, Diameter	AI	Artificial Intelligence
AKIEC	Actinic Keratoses and Intraepithelial Carcinoma	AUC	Area Under the Curve
BCC	Basal Cell Carcinoma	BKLs	Benign Keratosis-like Lesions
CAD	Computer-Aided Detection	CNNs	Convolutional Neural Networks
COCO	Common Objects in Context	DCNNs	Deep Convolutional Neural Networks
DF	Dermatofibroma	EIS	Electrical Impedance Spectroscopy
F1	F1 Score	Gradio	Gradio (Python Package)
HAM	HAM10000 Dataset	ISIC	International Skin Imaging Collaboration
MEL	Melanoma	MSI	Multispectral Imaging
NDOELM	Non-destructive Online Ensemble Learning Method	NMSC	Non-Melanoma Skin Cancer
NV	Melanocytic Nevi	PC	Personal Computer
PSNR	Peak Signal-to-Noise Ratio	RCM	Reflectance Confocal Microscopy
ResNet	Residual Network	RGB	Red, Green, Blue
ROC	Receiver Operating Characteristic	SGD	Stochastic Gradient Descent
SSIM	Structural Similarity Index Measure	SVM	Support Vector Machine
VASCs	Vascular Lesions	VGG	Visual Geometry Group
